# A qualitative inquiry of speech-language therapists’ views about breastfeeding management

**DOI:** 10.4102/sajcd.v72i1.1070

**Published:** 2025-01-21

**Authors:** Danica Schlome, Esedra Krüger, Bhavani Pillay

**Affiliations:** 1 Department of Speech-Language Pathology and Audiology, Faculty of Humanities, University of Pretoria, Pretoria, South Africa

**Keywords:** breastfeeding management, perspectives, speech-language therapists, scope and roles, qualitative research

## Abstract

**Background:**

Management of oropharyngeal dysphagia within the first few days of an infant’s life results in favourable breastfeeding outcomes, indicating the importance of investigating the breastfeeding management practices of speech-language therapists (SLTs) working within this field.

**Objectives:**

Little has been published about SLTs’ management of breastfeeding in low- and middle-income settings. This study explores the perspectives of a group of experienced South African SLTs on their approach to breastfeeding management.

**Method:**

Qualitative data were gathered through semi-structured online interviews with 12 experienced SLTs and were subjected to thematic analysis.

**Results:**

Three main themes emerged: (1) Approach to breastfeeding management; (2) Exposure, skills and knowledge related to breastfeeding management; and (3) Perspectives and attitudes towards breastfeeding management. Participants demonstrated a clear understanding of their roles within the scope of breastfeeding management, which aligns with established literature. Their approaches appeared to be influenced by their perspectives and attitudes towards breastfeeding, as well as their exposure, skills and knowledge in this area. The study underscores the need for breastfeeding training in undergraduate programmes and highlights the demand for continuous professional development opportunities.

**Conclusion:**

While the findings are drawn from a small sample of experienced clinicians, they offer valuable insights for speech-language pathology clinical educators and professional organisations.

**Contribution:**

This study suggests a re-evaluation of university curricula to enhance exposure to breastfeeding management.

## Introduction

Globally, research has proven that exclusive breastfeeding (EBF) is the best source of nutrition for infants and can be defined as a form of preventative medicine (Jama et al., 2020; Moukarzel et al., 2020). Various health benefits are associated with EBF. Infants gain both physiological and cognitive advantages from EBF, in addition to obtaining adequate nutrition (Jama et al., 2020; WHO, 2024). Healthcare professionals, including speech-language therapists (SLTs), should receive adequate training to promote EBF both within healthcare facilities and beyond. Speech-language therapists possess the potential to exert influence on breastfeeding maintenance, thus helping to prevent early breastfeeding cessation (Campbell et al., 2022). When faced with feeding difficulties, SLTs intervene to promote safe, functional and efficient feeding, consequently reducing aspiration risk and improving overall quality of life (Arvedson et al., 2020).

Given their pivotal role in providing swallowing and feeding intervention to neonates, it is imperative that SLTs have access to theoretical and clinical training of the highest standard. Speech-language therapists need to receive training in typical infant swallowing and feeding, as well as in the assessment and management of oropharyngeal dysphagia (OPD; ASHA, 2016; Medeiros et al., 2017), as early intervention for OPD during breastfeeding is a core aspect of their role (Arvedson et al., 2020).

Speech-language therapists tend to be exposed to various difficulties experienced by the breastfeeding dyad. These include difficulties with latching, milk transfer and uncoordinated sucking, swallowing and breathing patterns in infants, in addition to medical conditions associated with feeding difficulties, including ankyloglossia, cleft lip and/or palate, hypoxic-ischaemic encephalopathy and a multitude of genetic conditions (Mahurin-Smith & Genna, 2018). The SLT therefore plays an important part in the management of these difficulties, because of the direct link to OPD, in which SLTs are core role players (ASHA, 2016).

Speech-language therapists ensure adherence to evidence-based practice (EBP) and provide holistic support to families experiencing breastfeeding difficulties (Mahurin-Smith & Genna, 2018). Practice-based evidence (PBE), which complements EBP, helps bridge the gap between research findings and clinical application by acknowledging the significance of clinical decision-making (Crooke & Olswang, 2015). Given the lack of established practice standards in breastfeeding management within this field, PBE could be particularly valuable for SLTs (Lemoncello & Ness, 2013).

Counselling, collaboration with parent-neonate dyads, and early OPD management within the first few days of life lead to favourable breastfeeding outcomes, underscoring the importance of appropriate breastfeeding management education for SLTs (Kritzinger et al., 2024; Medeiros et al., 2017). Collaboration between SLTs and International Board-Certified Lactation Consultants (IBCLCs) represents an ideal combination of skills for holistic care (Mahurin-Smith & Genna, 2018), ensuring safe and efficient swallowing and feeding, thus promoting optimal nutrition for infants to thrive (Arvedson et al., 2020).

A study evaluating SLTs’ protocols for assessing breastfeeding highlighted the scarcity of validated, standardised protocols in use, which has implications for breastfeeding management (De Oliveira et al., 2019). An unpublished survey conducted in South Africa revealed that the role description of SLTs within breastfeeding teams was unclear, indicating a need for further research, thus justifying the present study (Eksteen et al., 2020).

Research investigating South African SLTs’ perspectives on breastfeeding management is crucial to ensure that clinicians are well-equipped to provide evidence-based intervention. Such efforts can lead to infants receiving sufficient nutrition and potentially enjoying the benefits associated with EBF, while also enhancing mothers’ physiological and emotional well-being (Brahm & Valdés, 2017). By identifying gaps in this field, SLTs’ training needs can be evaluated and addressed accordingly. The present study aims to explore the perspectives of a sample of SLTs working in South Africa regarding their breastfeeding management practices.

## Research methods and design

### Study design

A descriptive, qualitative research design was used to collect data through semi-structured, online interviews.

### Study population and sampling

Potential participants were recruited using an infographic, circulated on professional social media groups and through the researchers’ network. Prospective participants who volunteered were contacted to confirm the inclusion criteria by the researcher, the first author. Speech-language therapists were eligible for inclusion if they were rendering services to infants and families with breastfeeding difficulties in a clinical setting, were registered with the Health Professions Council of South Africa and had at least 5 years of experience. Maximum variation sampling, a purposive method, was utilised, allowing the selection of SLTs frequently exposed to breastfeeding management, with varied work settings (e.g. government-funded or private), work contexts (e.g. clinic, hospital or academia), years of experience and places of study (Pope & Mays, 2020).

Twelve qualified SLTs had varying years of experience, ranging from 5 to more than 10 years, with half the participants (*n* = 6) having 6–10 years of experience. Participants obtained undergraduate qualifications from several universities across South Africa, with half having postgraduate degrees. Some participants completed (*n* = 2) or are completing (*n* = 2) additional training, including the South African Certified Lactation Consultant course, neuro-developmental therapy training (*n* = 3), Sequential-Oral-Sensory Approach to Feeding (*n* = 1) and additional breastfeeding-related seminars (*n* = 9). Participants work across various contexts, with some working in multiple contexts, including regional hospitals (*n* = 1), academic hospitals (*n* = 3), private hospitals (*n* = 5), private practices (*n* = 6), universities (*n* = 3) and schools (*n* = 1).

### Data collection

Data were collected by one qualified SLT, via Microsoft Teams, with interviews lasting approximately 45 min. A self-compiled interview schedule (see Online Appendix 1) based on previous studies guided discussions (Blake, 2014; Eksteen et al., 2020).

### Data analysis

Interviews were transcribed manually and coded using ATLAS.ti software. Data were analysed qualitatively, using thematic analysis (Brink et al., 2018).

### Ethical considerations

Institutional ethical approval was obtained from the University of Pretoria Faculty of Humanities Research Ethics Committee (reference no.: HUM002/1221) and written informed consent was requested and obtained from all participants prior to data collection.

## Results

Findings are described according to three themes that were identified (Table 1).

**TABLE 1:** Three identified themes and descriptions.

Themes	Description
Approach to breastfeeding management	Participants identified their perceived roles and specific practices involved
Exposure, skills and knowledge towards breastfeeding management	Participants explained clinical experience with breastfeeding management, including various difficulties that they encountered and recommendations for improving clinical competence
Perspectives and attitudes towards breastfeeding management	Participants reviewed their level of confidence in managing breastfeeding, as well as the interplay between the roles of SLTs and lactation consultants

SLTs, speech-language therapists.

### Theme 1: Speech-language therapists’ approach to breastfeeding management

Participants’ perspectives on their approach to breastfeeding management, included views on assessment as well as management and collaboration. Participants indicated various assessment areas, assessment tools and treatment areas perceived to be part of their role in breastfeeding management. All participants indicated performing a clinical bedside swallow evaluation, including a comprehensive case history, assessment of feeding-specific areas and an oral motor assessment, to exclude structural abnormalities, like tongue-ties, lip-ties or cleft lip and/or palate (CL/P), in addition to assessing the functionality of muscles, cranial nerves and reflexes involved in breastfeeding. Practitioners with clinical exposure to tongue- and lip-ties use the Assessment Tool for Lingual Frenulum Function (Hazelbaker, 2017) and the Kotlow Tongue-Tie Classification System (Kotlow, 1999). It appears that no standardised protocol is implemented.

A baseline evaluation is obtained through clinical assessment of non-nutritive sucking (NNS). Participants stated that further assessment of oral feeding is important to evaluate latching, sucking, feeding endurance and swallow physiology. Participants focus on hyolaryngeal elevation and excursion, through swallow palpation; observation of swallowing phases and signs of aspiration; and infants’ ability to coordinate sucking, swallowing and breathing, adding to the quality and length of feeds. Participants explained that infants’ vitals are often monitored, and stress signs, level of alertness and interaction with mothers are observed. It appears as though participants did not mention observations of postural adaptations or specific components of gross motor movement in their assessment.

Eight SLTs reported that their evaluation protocol involves self-developed checklists, setting-specific assessments and qualitative interviewing and observation, while few SLTs use standardised assessments, including the Neonatal Feeding Assessment Scale (Viviers et al., 2016), the Preterm Infant Breastfeeding Behaviour Scale (Nyqvist et al., 1996) and the Neonatal Eating Outcome Assessment (Pineda et al., 2016). Only one SLT mentioned recommending a modified barium swallow if patients experience signs of OPD.

Following the assessment, participants shared that they play a role in treating various breastfeeding aspects. Participants stated that their treatment occurs as a part of a team through collaboration with other professionals, ensuring holistic case management of breastfeeding dyads, especially when complex conditions are involved. These conditions include genetic and cardiac disorders, respiratory diseases, structural anomalies, colic and the effect of medication on breastfeeding. Many participants reported that ‘managing mothers’ expectations of breastfeeding’ is important in achieving realistic outcomes, contributing to maternal well-being. Participants expressed their view that positive maternal well-being lends itself to an optimal environment fostering the development of successful bonding between parents and infants. Speech-language therapists may collaborate with psychologists, ensuring reduced parental stress, and enhancing breastfeeding capacity and efficiency. One participant explained that a parents’ group in the neonatal intensive care unit (NICU) provided support, reducing maternal breastfeeding-related stress. Participants reported that collaboration with mothers may provide opportunities to highlight the importance of Kangaroo Mother Care and identification of infant stress cues during feeding. Clinicians in the sample felt that parents’ ability to read stress cues assists in infant state regulation and parents’ ability to identify the optimal state – quiet-alert – for feeding. Two SLTs train mothers in monitoring infant feeding success by encouraging recording of the time their infant spent on the breast at each feed, promoting the breastfeeding parents’ autonomy. Parents may also be counselled by SLTs regarding paced feeding and different feeding methods, if infants are not yet able to gain sufficient nutrition directly from the breast.

Five participants expressed that determining infants’ ideal feeding method is an important role if direct breastfeeding is not immediately indicated. This includes transitioning from tube to oral feeding, or using syringes or cups, based on the integrity of suck-swallow-breathe (SSB) coordination. Participants reported involvement in supplementation of feeding methods, collaborating with dietitians, and ensuring infants receive optimal nutrition for weight gain and development. Participants appeared to be involved in OPD management predominantly and less in general breastfeeding practice.

From the sample’s perspective, SLTs fulfil roles in providing somatic-oral stimulation, supplying tactile and proprioceptive input to infants with sucking difficulties in preparation for feeding. Speech-language therapists may collaborate with occupational and physical therapists to assist in infant positioning prior to feeding. Specific aspects that SLTs perceive to be their role include facilitation of an appropriate latch, ensuring adequate sucking and swallowing, and monitoring feeding endurance for successful milk extraction. Where appropriate, participants are involved in providing adaptive methods, like nipple shields.

Participants explained they perceive the SLT’s role in breastfeeding to involve medically complex infants, or infants with abnormal oral features, namely CL/P, high palatal arches and tongue- and/or lip-ties. One SLT with clinical exposure to these anomalies expressed that her role includes ‘identification of these structural anomalies, and precise intervention, pre- and post-operatively, for the medically-complex infant population’. Collaboration with other professionals including maxillofacial and plastic surgeons, otorhinolaryngologists, paediatricians, neonatologists, paediatric cardiologists, paediatric dentists, geneticists, nurses and occasionally, chiropractors was viewed as essential.

In certain instances, participants may work with lactation consultants, or if trained as lactation consultants themselves, may incorporate lactation principles into SLT breastfeeding intervention. These include educating mothers about hand expression massage and discussing breastfeeding logistics when mothers return to work.

### Theme 2: Speech-language therapists’ exposure, skills and knowledge

Participants reported that knowledge obtained from their SLT university training emphasised support for neonates’ swallowing and feeding when managing paediatric dysphagia. Anatomy and physiology of swallowing and pathophysiology of aspiration appeared to be the focus of participants’ university training. Clinicians in the sample expressed that university training equipped them with knowledge and skills to ‘manage common difficulties experienced by mothers and infants during breastfeeding’. These difficulties include the management of medically complex cases in which infants exhibit signs of OPD, because of medical conditions like hypoxic-ischemic encephalopathy, traumatic brain injury, neonatal jaundice, Trisomy 21, epilepsy, CL/P, tongue- and/or lip-ties, laryngomalacia, Pierre Robin Sequence, low birth weight and preterm birth, respiratory conditions, gastroesophageal reflux disease, allergies, and intolerances. However, one SLT in the sample felt that in-depth training on managing breastfeeding difficulties practically is required at an undergraduate level:

As far as I remember, I could be wrong, but based on my memory, I don’t think that we had any lectures on breastfeeding and if we did then maybe it was just mentioned on the side. (n.p)

Participants explained that mothers encounter breast-related difficulties, involving pain, bleeding nipples, mastitis, flat or inverted nipples, plugged ducts and engorgement. Occasionally, the let-down reflex is delayed, or milk supply is poor, and difficult labour and birth were seen as contributing to breastfeeding difficulties, as reflected in the following: ‘…traumatic birth experiences, mothers suffer from stress and anxiety related to breastfeeding, leading to postnatal depression’.

Common difficulties treated by participants involve infant positioning, latching, and refusal of the breast. Participants also experienced the following challenges with infants: poor alertness, inadequate labial seal, poor SSB coordination, immature sucking, poor endurance, poor milk extraction and transfer, and ultimately poor weight gain.

Although many participants felt fairly competent in managing breastfeeding difficulties clinically, they could not solely rely on their university training, as ‘more theoretical knowledge, more clinical exposure during undergraduate training and continuing professional development (CPD) courses with a practical component’ are necessary to improve competence in breastfeeding management. Independent research, use of training videos, and joining breastfeeding social media groups, appeared to foster competence in breastfeeding management among the sample.

Many participants in the study expressed a need for improved training on breastfeeding management, theoretically and practically, emphasised by their own attendance of breastfeeding-related courses and hands-on experience following graduation. It was suggested that newly qualified SLTs attend CPD courses related to breastfeeding management, ‘with a practical component, so you get to practise the skills’, thereby enhancing existing skills. Participants felt that familiarity and increased exposure to the NICU, and experience with paediatric inpatients may assist in fostering confidence in providing breastfeeding-related services. Some participants expressed that competence was enhanced by observing colleagues and engaging in clinical case discussions, following observations in the NICU and paediatric wards. Participants felt that developing a mentoring system between newly qualified SLTs and experienced clinicians may improve the clinical skills of less experienced professionals. This was reiterated by the following: ‘The moment you are mentored and shown how to do it, it becomes more of a practical thing’. Some participants showed a lack of knowledge regarding the role, and scope of practice of lactation consultants, to which they alluded further training is required, to ‘learn more about the lactation consultant’s scope’.

### Theme 3: Speech-language therapists’ attitudes towards breastfeeding management

Overall, the sample portrayed positive attitudes towards the management of breastfeeding. One participant mentioned they ‘find breastfeeding management extremely interesting’ and it is evident from responses that breastfeeding management can be ‘a bit more [*clinically*] challenging’, which this sample appeared to enjoy.

Participants trained as lactation consultants offered insight into the perceived role of lactation consultants during breastfeeding management and the interplay with the SLT’s role. According to participants, lactation consultants problem-solve difficulties experienced by breastfeeding dyads, not solely related to medical conditions affecting the oral or pharyngeal swallowing phases in infants. These include problems with the breastfeeding method, hormonal difficulties related to milk supply, expression, colostrum and engorgement. Lactation consultants may guide mothers about long-term breastfeeding plans and provide counselling. Participants felt that, in contrast to SLTs, lactation consultants do not intervene in pathological feeding, which includes difficulties related to structural or neurological disorders that may cause difficulty breastfeeding, defining the SLT’s scope and stating the SLT’s importance in breastfeeding management.

Participants reported feeling less competent about certain breastfeeding management areas, exacerbated by diverse cultural and linguistic landscapes in work contexts. Many difficulties that SLTs in the study encountered also involve ‘breast-related issues’, problems with milk supply and infants who refuse the breast, portraying aversive behaviour. Participants also did not feel competent with difficulties solely affecting mothers or affecting infants who do not have medical risk factors, like, infants born full term with a normal birth weight. The interplay between SLTs’ and lactation consultants’ roles and the associated complementary functions was apparent.

## Discussion

**FIGURE 1:**
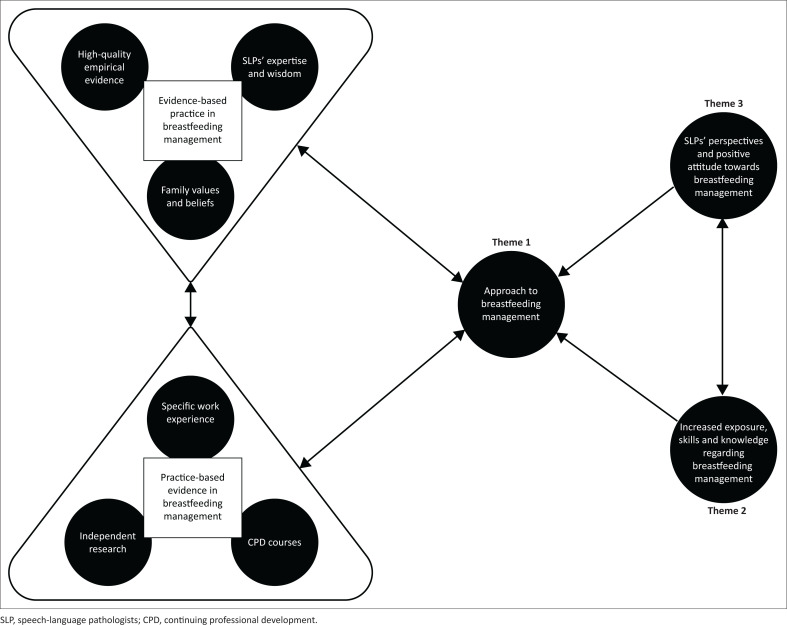
Developing evidence: Related themes of breastfeeding management practices.

Most participants in the study had between 6 and 10 years of experience and frequently worked with breastfeeding dyads, indicating a knowledgeable sample. They demonstrated an awareness of their role as SLTs in breastfeeding management, although their acquisition of skills and knowledge seemed to stem from internal motivation and a passion for their work. These findings align with similar research, suggesting that SLT graduates may not always feel comfortable providing intervention to breastfeeding dyads without additional training (Mahurin-Smith, 2018). Participants in the current study expressed a positive attitude and interest in furthering their understanding of breastfeeding independently. A visual representation illustrating the themes that emerged and the relationships between them in the present study is provided in Figure 1.

Participants shared their views on skills and knowledge in breastfeeding management gained from their own self-guided training, working experience and CPD, which may have been influenced by their perspectives and positive attitudes (Figure 1). These aspects appear to inform SLTs’ approach to breastfeeding management, further enhanced by the application of EBP and PBE.

Evidence-based practice involves incorporating SLTs’ expertise and wisdom, high-quality empirical evidence, and the values and beliefs of breastfeeding dyads (Grove & Gray, 2018). To complement EBP, PBE in breastfeeding management includes specific work experience, independent research and breastfeeding-related CPD courses, thus improving SLTs’ ability to provide effective services to diverse breastfeeding dyads (Lemoncello & Ness, 2013).

**FIGURE 2:**
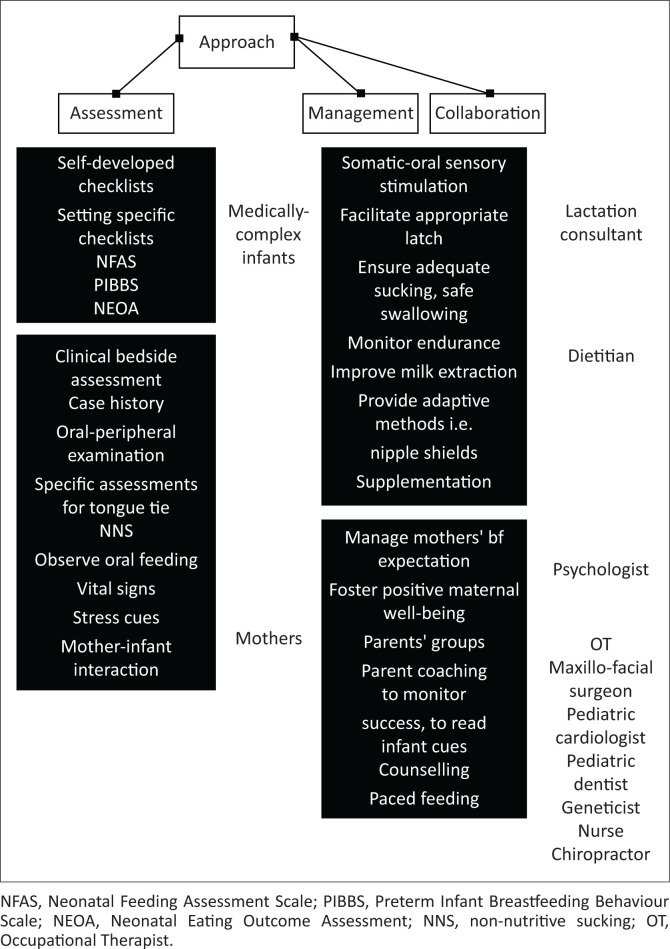
Participants’ approach to breastfeeding management.

Assessment of breastfeeding dyads serves as the starting point for management (Figure 2). As indicated by well-known literature regarding swallowing and feeding management, the sample of SLTs in this study typically begins with a clinical bedside swallow evaluation, including an oral motor examination, to identify functional and structural deficits affecting breastfeeding (Arvedson et al., 2020). Assessment allows clinicians to hypothesise the possibility of patients presenting with OPD. Non-nutritive sucking was also commonly assessed. Although NNS does not predict successful nutritive feeding, it provides a broad indication of sucking strength, sucking bursts, tongue cupping and integrity of the labial seal – prerequisites to oral feeding readiness (Pineda et al., 2019).

South African SLTs often encounter limited human and material resources in their working environments, which explains the limited use of formal assessments. Clinicians in this sample demonstrated resourcefulness by utilising self-compiled checklists and setting-specific assessment protocols (Figure 2). An exception was the Neonatal Feeding Assessment Scale (Viviers et al., 2016), specifically developed for South Africa and similar contexts. The use of informal assessment procedures aligns with previous research, in which a sample of South African SLTs commented on the lack of breastfeeding management resources (Eksteen et al., 2020). The use of informal assessments and checklists appears to be common practice for SLTs working with paediatric dysphagia in low- and middle-income settings (De Oliveira et al., 2019). Instrumental assessment remains the gold standard for diagnosing OPD and aspiration (Lo Re et al., 2019), but only one participant alluded to using instrumental assessment. It is evident that a lack of standardised assessment protocols and guidelines exist, which warrants further research.

Participants demonstrated an interest in interprofessional collaboration, ensuring holistic treatment of breastfeeding dyads with complex medical needs (Figure 2). This practice aligns with breastfeeding management research, highlighting collaboration as a determining factor of breastfeeding success (Mahurin-Smith & Genna, 2018).

An interest in and the need to be equipped was evident among participants; however, a lack of knowledge following their undergraduate training appeared to be a barrier to competency in this field. Successful management of breastfeeding difficulties by participants depended on their exposure, skills and knowledge, both theoretical and practical. Additionally, participants’ interests and opinions motivated them to pursue further training in breastfeeding management, thereby enhancing their confidence (Figure 1). Increasing SLTs’ exposure to the treatment of breastfeeding difficulties during university training may enable clinicians to focus on building skills and knowledge. This could positively impact SLTs’ approaches to breastfeeding management, shaping their perspectives and attitudes to foster confidence and competence when treating breastfeeding dyads. Inadequate competence and a lack of confidence may lead SLTs to provide insufficient service delivery to vulnerable populations in South Africa. University training programmes are urged to re-evaluate their curricula to better respond to the contextual needs of clinicians and patients. This is critically important in South Africa and other low-resourced settings, where qualified allied health professionals, such as SLTs, are scarce.

Participants showed positive attitudes towards breastfeeding management and their role in breastfeeding teams. They believe that an improved understanding of the lactation consultant’s role would assist in efficacious treatment, allowing patients to reap the benefits offered by the two professions. This finding correlates with previous research (Mahurin-Smith & Genna, 2018).

Although SLTs appear to perform roles within breastfeeding teams in accordance with the literature, a lack of interprofessional collaboration with lactation consultants results in SLTs having less confidence, working in isolation or completing lactation courses to address their knowledge needs. As this is not a possibility for all SLTs, improved opportunities for interprofessional collaboration and education remain critical. Speech-language therapists are key members of breastfeeding teams and work with other specialists to manage the complexity of breastfeeding difficulties. Speech-language therapists require a specific knowledge base and skill set, which may be addressed in CPD and mentoring programmes. Professional bodies are ideally situated as catalysts for learning opportunities and could transform SLTs’ services to diverse families.

### Limitations

This study purposefully included participants interested in breastfeeding management and findings present a specialist population’s perspective, which does not include all SLTs dealing with breastfeeding difficulties daily. Perspectives of inexperienced and recently graduated individuals may contribute to the continuing improvement of training for SLTs and further research is necessary. The small sample size may not indicate generalisable results although the findings are valuable.

## Conclusion

The study provides a detailed description of the unique role of experienced SLTs in managing breastfeeding difficulties. Speech-language therapists often become involved in the care of infants with OPD. It appears that standardised assessment and treatment protocols are not utilised in this sample. The findings suggest that greater exposure to breastfeeding management during university education, along with additional professional development opportunities, could enhance SLTs’ skills, knowledge and confidence in this area. Moreover, clarifying scopes of practice, ensuring access to mentoring, fostering confidence and increasing opportunities for interprofessional collaborative practice among members of the breastfeeding team are emerging needs for SLTs working with breastfeeding dyads. These findings hold potential value for both SLT researchers and educators.
